# Seasonal Variations in the Risk of Outpatient Acute Kidney Injury in Patients with Chronic Kidney Disease

**DOI:** 10.3390/diagnostics16060845

**Published:** 2026-03-12

**Authors:** Hiroyuki Nakanoh, Kenji Tsuji, Kazuhiko Fukushima, Naruhiko Uchida, Soichiro Haraguchi, Shinji Kitamura, Jun Wada

**Affiliations:** 1 Department of Nephrology, Rheumatology, Endocrinology and Metabolism, Graduate School of Medicine, Dentistry and Pharmaceutical Sciences, Okayama University, Okayama 700-8558, Japan; bambubu2756@gmail.com (H.N.);; 2 Department of Nephrology, Aoe Clinic, Okayama 700-0941, Japan

**Keywords:** acute kidney injury, chronic kidney disease, outpatients, seasons

## Abstract

**Background/Objectives**: Acute kidney injury (AKI) frequently occurs in the outpatient setting and is associated with adverse renal and survival outcomes. However, there is no established definition of outpatient AKI, and the risk factors, especially seasonal variation, remain limited. This study aimed to investigate seasonal variation in the risk of outpatient AKI. **Methods**: This retrospective observational study used routinely collected clinical laboratory data from a single hospital in Japan between 2007 and 2022. Outpatient AKI was defined as ≥35% relative decline in estimated glomerular filtration rate (eGFR) compared with a preceding outpatient measurement obtained within 14–90 days. Monthly and seasonal variations in outpatient AKI risk in patients with chronic kidney disease (CKD) were evaluated using logistic regression models. Subgroup analyses were performed according to AKI stage, age group, and CKD stage. **Results**: A total of 203,853 outpatient records were analyzed. The incidence of outpatient AKI was highest in August and lowest in November. Analyses demonstrated significantly increased odds ratios of outpatient AKI in January, February, July, and August. Seasonally, the risk was significantly higher during the summer. Stage-specific analyses showed that AKI stage 1 was more frequent in the summer, whereas AKI stage 2 tended to increase during the winter. **Conclusions**: Outpatient AKI exhibits distinct seasonal patterns, with increased risk during both summer and winter and differential associations according to AKI severity and baseline kidney function. Recognition of these patterns may help identify vulnerable populations and inform targeted preventive strategies for outpatient AKI.

## 1. Introduction

Acute kidney injury (AKI) is an increasingly common global clinical condition, with its incidence rising steadily as a major public health concern, largely driven by population aging, the growing prevalence of lifestyle-related diseases, and the increasing complexity of medical care [1,2]. To date, numerous studies have primarily investigated hospital-acquired AKI (HA-AKI), reporting its association with substantial adverse outcomes, including increased mortality, prolonged hospital stay, progression to chronic kidney disease (CKD), and higher healthcare costs [3,4,5,6,7]. In recent years, community-acquired acute kidney injury (CA-AKI), which occurs in the outpatient setting, has been reported to be more common than HA-AKI and is associated with adverse renal outcomes and increased mortality [8,9,10,11,12,13]. Outpatient AKI without subsequent hospitalization has also been shown to be associated with an increased risk of end-stage kidney disease and mortality [14]. Furthermore, the even mildest form of outpatient AKI (1.5–1.9 times the baseline creatinine) has been reported to significantly increase the risk of mortality [8]. Outpatient AKI occurred in 1.4–2.4% of patients, compared with only 0.3% for hospital-acquired AKI, indicating that a substantial proportion of AKI cases occur in the outpatient setting and are initially managed by outpatient healthcare providers [8,15]. Therefore, identifying the risk factors for CA-AKI, particularly outpatient AKI without subsequent hospitalization, is of critical importance for early detection and prevention. Previous studies have reported that risk factors for CA-AKI include recent episodes of vomiting, diarrhea, and urinary retention, as well as male sex, the use of medications such as nonsteroidal anti-inflammatory drugs (NSAIDs), renin–angiotensin–aldosterone system (RAAS) blockers, and diuretics [12,16]. In addition, comorbid conditions such as diabetes, hypertension, CKD, heart failure, liver disease, and smoking have been identified as important predisposing factors [9,16]. Unlike HA-AKI, outpatient AKI may also be influenced by environmental factors, among which seasonal variation represents a potentially important but less well-characterized determinant. Seasonal variation has been recognized as an important determinant of disease incidence in various medical conditions, including AKI [17]. Several studies have reported seasonal patterns in CA-AKI, with an increased risk during the winter months [18,19]. However, data on seasonal variation in outpatient AKI, particularly in cases not requiring hospitalization, remain limited. Understanding seasonal variation in outpatient AKI in a nephrology outpatient setting is important for optimizing patient education and guiding timely interventions during high-risk periods. In this retrospective observational study, we aimed to investigate whether seasonal variation influences the risk of outpatient AKI in a nephrology outpatient population, primarily consisting of patients with underlying CKD.

## 2. Method

### 2.1. Data Source

This study is a retrospective observational study using routinely collected clinical data. Data were obtained from the clinical laboratory database of the Department of Nephrology at Okayama University Hospital (Okayama, Japan) during the study period from November 2007 to December 2022. The database includes patient demographics (age and sex), dates of outpatient testing, and serum creatinine–based estimated glomerular filtration rate (eGFR) values. eGFR was calculated using the Japanese Society of Nephrology equation. The study protocol was approved by the ethics committee of Okayama University Hospital Institutional Review Board, Okayama, Japan (approval number: 1908-022, 2305-018), and conducted in adherence with the tenets of the Declaration of Helsinki. Informed consent from individual patients was waived because all data were anonymized for research purposes.

### 2.2. Study Population and Cohort Definition

**Figure 1:**
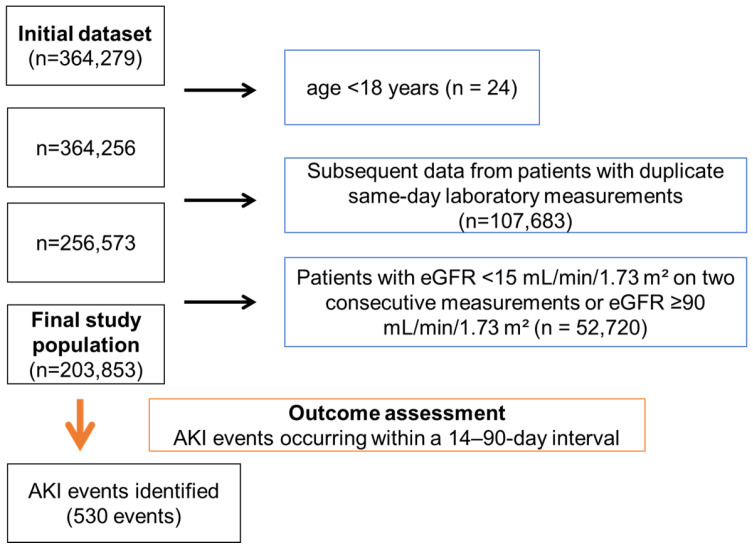
Flow diagram of study population selection. eGFR: estimated glomerular filtration rate; AKI: acute kidney injury.

A total of 364,279 records were extracted (Figure 1). Patients aged <18 years were excluded from the analysis. The dataset included patients undergoing maintenance hemodialysis or peritoneal dialysis. Because hemodialysis patients generally undergo blood tests twice on the same day (before and after dialysis), when two laboratory measurements were recorded on the same date, only the first measurement was retained and all subsequent measurements were excluded. To evaluate patients with CKD, records from patients with an eGFR ≥ 90 mL/min/1.73 m^2^ were excluded. In addition, records from patients who exhibited an eGFR < 15 mL/min/1.73 m^2^ on two consecutive measurements were also excluded. Although this approach does not allow for the direct identification and exclusion of patients undergoing peritoneal dialysis, it is expected to exclude the majority of such patients. CKD stages were defined according to the KDIGO guidelines as follows: stage 1, eGFR ≥ 90 mL/min/1.73 m^2^; stage 2, eGFR 60–89 mL/min/1.73 m^2^; stage 3, eGFR 30–59 mL/min/1.73 m^2^; stage 4, eGFR 15–29 mL/min/1.73 m^2^; and stage 5, eGFR < 15 mL/min/1.73 m^2^ [20].

### 2.3. Outcome Measures

Because there is no uniform definition of CA-AKI, the timing and magnitude of its assessment vary substantially across previous studies, and no standard definition has been established for detecting AKI in the outpatient setting. In this study, the primary outcome, outpatient AKI, was defined as a ≥35% relative decline in eGFR, corresponding to a ≥1.5-fold relative increase in serum creatinine, compared with a preceding outpatient eGFR measurement. As secondary outcomes, AKI stages 1, 2, and 3 were determined according to established guidelines and were defined as relative declines in eGFR of 35–49.9%, 50–74.9%, and ≥75%, respectively, compared with the preceding laboratory value [21]. Outpatient AKI was defined only when a preceding laboratory measurement was available within 14 to 90 days before the index test. This time window was selected to reflect routine outpatient follow-up intervals, as serum creatinine measurements in outpatient settings are typically obtained at intervals of several weeks to months rather than within the 48 h to 7 days specified in the KDIGO criteria. This approach has been adopted in several previous studies investigating outpatient or CA-AKI. If no laboratory data were available within this time window, the event was not classified as outpatient AKI. We plotted the number and proportion of patients with outpatient AKI by month and identified the months with the highest and lowest proportions of patients with AKI. The seasons were defined as spring (March–May), summer (June–August), autumn (September–November), and winter (December–February). November and autumn were selected as the reference categories because they exhibited the lowest proportions of patients with outpatient AKI.

### 2.4. Statistical Analysis

Data were summarized as percentages for categorical variables and means ± standard deviation (SD). Monthly and seasonal variations in the risk of outpatient AKI were evaluated using logistic regression models in patients with CKD. Odds ratios (OR) and 95% confidence intervals (CI) were calculated to estimate the relative odds of AKI for each month and season, with November and autumn used as the respective reference categories. Error bars indicate 95% confidence intervals. All statistical analyses were performed using JMP version 18 (SAS Institute Inc., Cary, NC, USA). All *p*-values were calculated as two-sided. Significance was defined as *p*-values <0.05.

## 3. Result

### 3.1. Patient Characteristics

The baseline characteristics of patients included in this 15-year observational study (2007–2022) are shown in Table 1. A total of 203,853 outpatient records were analyzed. Of these, 57.63% were male, and the mean age was 62.9 ± 14.9 years. The mean eGFR was 51.3 ± 20.4 mL/min/1.73 m^2^, indicating that a substantial proportion of the study population consisted of patients with CKD.

### 3.2. Seasonal and Monthly Variations in Outpatient AKI and Renal Parameters

**Scheme 1:**
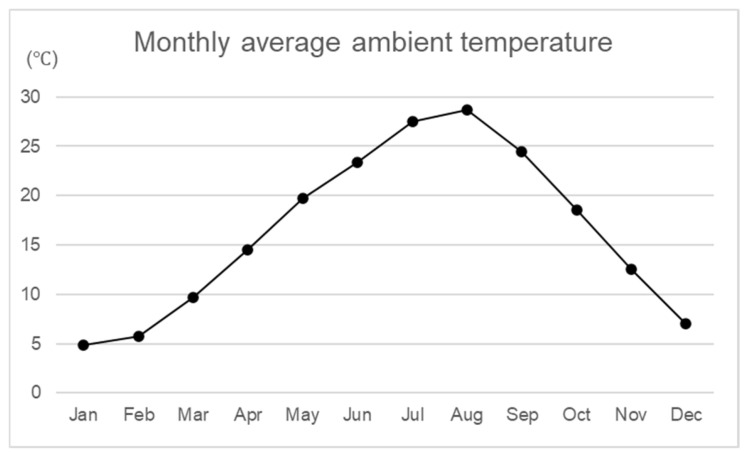
Monthly average ambient temperature in Okayama, Japan. Data represent monthly mean temperatures across the study period in Okayama, Japan (2007–2022), based on data from the Japan Meteorological Agency.

**Figure 2:**
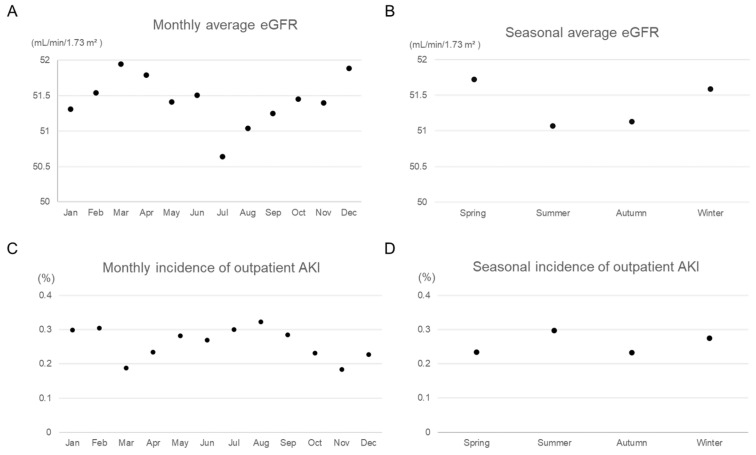
Average eGFR and Incidence of Outpatient AKI. (**A**) Monthly variation in the average estimated glomerular filtration rate (eGFR). (**B**) Seasonal variation in the average eGFR. (**C**) Monthly incidence of outpatient acute kidney injury (AKI). (**D**) Seasonal incidence of outpatient AKI.

We first examined seasonal variations in ambient temperature in Okayama, Japan (2007–2022), based on data from the Japan Meteorological Agency. August exhibited the highest average ambient temperature, whereas January had the lowest (Scheme 1) [22]. The mean eGFR peaked in March and reached its lowest level in July, with seasonal eGFR values being lowest during the summer (Figure 2A,B). The incidence of AKI was highest in August and during the summer, whereas it was lowest in November and during the autumn (Figure 2C,D). These seasonal patterns in renal parameters were closely associated with seasonal variations in ambient temperature.

**Figure 3:**
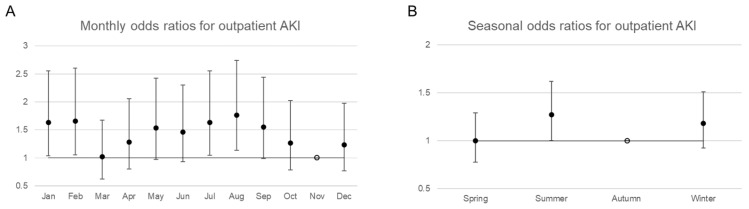
Odds Ratio for Incidence of Outpatient AKI. (**A**) Odds ratio for incidence of outpatient acute kidney injury (AKI) by month. (**B**) Odds ratio for incidence of outpatient AKI by season. October and Autumn were used as the reference season (odds ratio = 1.0).

In the month-based analysis, significantly increased odds of outpatient AKI were observed in January (OR, 1.62; 95% CI, 1.03–2.55), February (OR, 1.65; 95% CI, 1.05–2.60), July (OR, 1.63; 95% CI, 1.04–2.55), and August (OR, 1.76; 95% CI, 1.13–2.73), compared with the reference month (Figure 3A). In the seasonal analysis, the odds of outpatient AKI were significantly higher during the summer (OR, 1.27; 95% CI, 1.00–1.61), whereas a non-significant trend toward increased risk was observed during the winter (OR, 1.18; 95% CI, 0.92–1.50), compared with the reference season (Figure 3B).

### 3.3. Subgroup Analyses of Outpatient AKI by AKI Stage, Age, and CKD Stage

**Figure 4:**
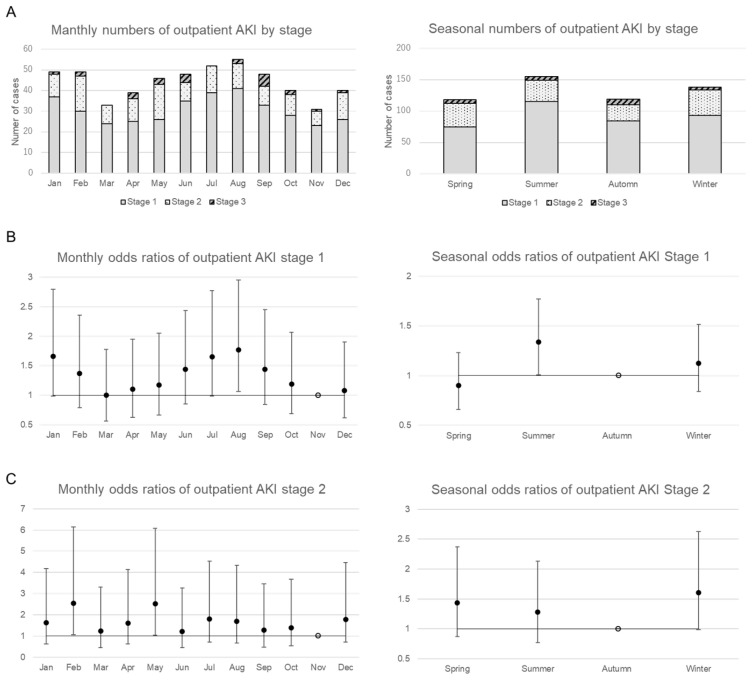
Monthly and Seasonal Patterns of Outpatient AKI by stage. (**A**) Incidences of outpatient acute kidney injury (AKI) by AKI stage. (**B**) Odds ratio for incidence of outpatient by AKI stage 1. (**C**) Odds ratio for incidence of outpatient AKI by AKI stage 2. October and Autumn were used as the reference season (odds ratio = 1.0).

For AKI stage 1, monthly analyses demonstrated peaks in January and August, with a significantly increased risk observed in August (OR, 1.77; 95% CI, 1.06–2.95) (Figure 4B). This pattern appeared to be closely associated with seasonal variations in ambient temperature. In the seasonal analysis, the risk of AKI stage 1 was significantly higher during the summer (OR, 1.33; 95% CI, 1.01–1.77) compared with the reference season. For AKI stage 2, monthly peaks were observed in February and May (Figure 4C). Although no statistically significant seasonal differences were detected, a trend toward increased risk was observed during the winter (OR, 1.60; 95% CI, 0.98–2.62). In contrast, no clear seasonal pattern was observed for AKI stage 3, likely due to the limited number of events.

**Figure 5:**
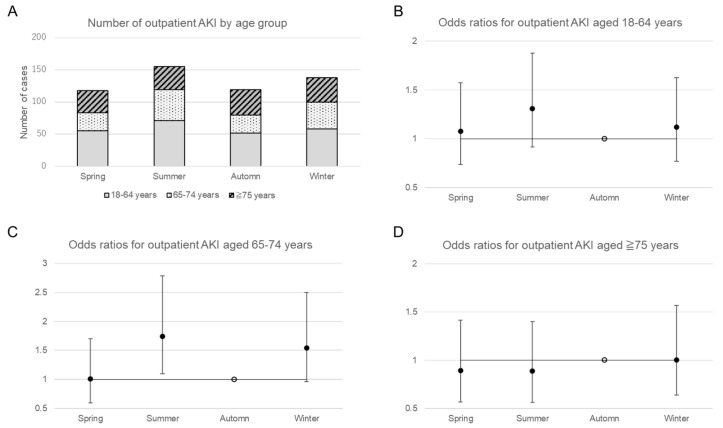
Odds Ratio for Incidence of Outpatient AKI by Age Group. (**A**) Incidences of outpatient acute kidney injury (AKI) stratified by age group. (**B**) Odds ratio for incidence of outpatient aged 18–64 years. (**C**) Odds ratio for incidence of outpatient aged 65–74 years. (**D**) Odds ratio for incidence of outpatient aged 75–99 years. Autumn was used as the reference season (odds ratio = 1.0).

Given these differences according to AKI severity, we next examined whether seasonal patterns of outpatient AKI varied by age. Age-stratified analyses demonstrated heterogeneous seasonal patterns of outpatient AKI risk. In patients aged 18–64 years, a higher risk of outpatient AKI was observed during the summer, although this increase did not reach statistical significance (Figure 5B). Among patients aged 65–74 years, the risk of outpatient AKI was significantly higher during the summer (OR, 1.77; 95% CI, 1.09–2.78), and a trend toward increased risk was also observed during the winter (OR, 1.54; 95% CI, 0.95–2.49) (Figure 5C). In patients aged 75 years and older, no significant seasonal differences in outpatient AKI risk were observed (Figure 5D).

**Figure 6:**
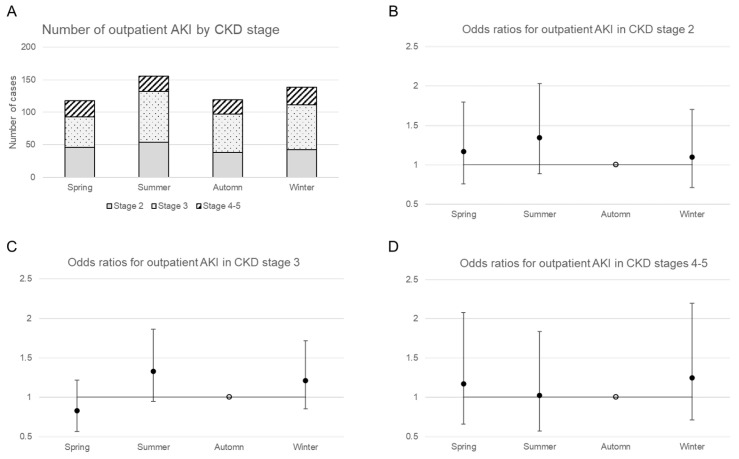
Odds Ratio for Incidence of Outpatient AKI by CKD stage. (**A**) Incidences of outpatient acute kidney injury (AKI) by chronic kidney disease (CKD) stage. (**B**) Odds ratio for incidence of outpatient AKI with CKD stage 2. (**C**) Odds ratio for incidence of outpatient AKI with CKD stage 3. (**D**) Odds ratio for incidence of outpatient AKI with CKD stage 4 or 5. Autumn was used as the reference season (odds ratio = 1.0).

To further explore the potential modifying effects of baseline kidney function, we next examined seasonal patterns of outpatient AKI according to CKD stage. Among patients with CKD stages 2 and 3, a trend toward an increased risk of outpatient AKI during the summer was observed (Figure 6B,C). In contrast, among patients with CKD stage 4 or higher, the risk of outpatient AKI did not increase during the summer (Figure 6D).

## 4. Discussion

The clinical importance of outpatient AKI has increasingly been recognized, as prior studies have demonstrated its association with higher risks of mortality and progression to end-stage kidney disease [8,9,10,11,12,13]. However, a standardized definition of outpatient AKI has not been established. With respect to the definition, many previous studies have adopted criteria corresponding to KDIGO Stage 1, such as a ≥50% increase in serum creatinine or an approximately ≥35% decline in eGFR [8,14,15]. In contrast, urine output criteria included in the KDIGO definition are rarely applicable in outpatient settings due to the practical difficulty of measuring urine volume outside the hospital. The temporal criteria used in outpatient studies also vary considerably. Whereas KDIGO defines AKI as occurring within 48 h to 7 days, outpatient investigations have employed substantially longer observation windows, ranging from 180 days to as long as 18 months [8,14,15]. For example, a multivariable logistic regression model predicting outpatient AKI, defined as a ≥50% increase in serum creatinine over an observation period of up to 18 months, demonstrated moderate discrimination, suggesting that this operational definition identifies clinically coherent events in the outpatient setting [15]. Nevertheless, the observation intervals have been arbitrarily determined across studies, and such extended time frames may capture a broader spectrum of kidney function changes rather than strictly defined acute injury. Conceptually, outpatient AKI may encompass classical acute injury occurring over hours to days, acute-on-chronic fluctuations particularly in patients with underlying CKD, and transient hemodynamic declines related to volume status or medication effects [9]. Thus, outpatient AKI should be considered a heterogeneous spectrum of kidney function deterioration.

In this study, we demonstrated that the risk of outpatient AKI increased during both the summer and winter seasons. In the secondary analyses, the risk of AKI stage 1 was increased during the summer, whereas AKI stage 2 showed a tendency toward a higher risk during the winter. Previous studies focusing on hospitalized patients with CA-AKI have reported that AKI incidence is highest during the winter and lowest during the summer [18,23,24,25]. In contrast, several epidemiological studies have also reported an increased risk of AKI during hot summer periods, with higher ambient temperatures being associated with an elevated AKI risk [17,26,27]. These apparent discrepancies among studies are likely attributable to differences in the study populations and environmental contexts, particularly whether AKI events occurred in outpatient or inpatient settings. Patients managed in the outpatient setting are more likely to experience mild AKI, whereas hospitalized patients tend to have more severe AKI requiring inpatient care. Indeed, previous reports focusing on hospitalized patients with CA-AKI have suggested that mild AKI (stage 1) is more common during the summer months, whereas more severe AKI (stage 2–3) occurs more frequently during the winter [18]. Our results align with prior studies of hospitalized populations and suggest that the seasonal pattern of outpatient AKI differs by AKI severity. Notably, the seasonal pattern of outpatient AKI was attenuated in patients with advanced CKD (stage ≥ 4), in whom baseline renal dysfunction and reduced renal reserve may outweigh the effects of seasonal environmental stressors. This finding suggests that seasonal vulnerability to AKI may be more pronounced in patients with preserved or moderately impaired kidney function.

The mechanisms underlying seasonal variation in AKI are likely multifactorial and may involve complex interactions among environmental stressors (such as temperature and humidity), seasonal fluctuations in infectious and acute disease burden, and host-related factors. From an environmental stress perspective, both heat exposure and cold exposure may influence renal hemodynamics through distinct physiological pathways. Humans have a tremendous capacity to produce sweat, with maximal sweat rates often approaching or even exceeding 2 L/h, making dehydration relatively common during heat stress because fluid losses from sweating are frequently not fully compensated by drinking, even when fluids are readily available [28,29]. During heat stress, activation of the sympathetic nervous system, the renin–angiotensin–aldosterone system, and vasopressin secretion is well recognized, leading to a reduction in renal blood flow [29]. In addition, high ambient temperatures have been associated with other renal risk factors, including an increased incidence of urolithiasis, which may further contribute to renal stress during the summer months [30]. Taken together, multiple mechanisms may plausibly contribute to the increased risk of AKI during periods of elevated temperature. Epidemiological evidence further supports the association between high temperature and AKI risk. A nationwide study from Korea reported that when ambient temperature exceeded 28.8 °C, the risk of AKI hospitalization increased by 23.3% for each 1 °C rise [31]. Similarly, an analysis from England demonstrated that peaks in AKI-related hospitalizations were temporally associated with heatwave alerts [27]. Moreover, such associations have been reported not only in warmer regions but also in northern climate settings, including Canada, where higher ambient temperatures were associated with an increased risk of AKI hospitalization [32]. These findings collectively suggest that elevated ambient temperature may represent an important environmental stressor contributing to seasonal variation in AKI incidence. Conversely, exposure to cold temperatures is associated with increased sympathetic activity, enhanced vascular tone, and elevations in blood pressure, all of which may contribute to hemodynamic stress on the kidney [33,34]. Compared with heat exposure, however, the evidence linking cold exposure to AKI remains relatively limited. A nationwide study from Korea reported that cold exposure was associated with an increased risk of AKI and AKI-related mortality [35]. In addition, several population-based studies from different countries have demonstrated seasonal increases in AKI incidence and mortality during winter months [18,23,24,36]. Although the underlying mechanisms remain incompletely understood, these observations suggest that cold-related physiological stress and seasonal increases in acute illnesses may contribute to the heightened risk of kidney injury and adverse outcomes observed during colder periods.

AKI often develops secondary to other acute illnesses, such as cardiovascular disease or infection, and therefore may reflect the seasonality of the primary diagnosis [17,18]. This seasonal pattern of increased AKI incidence during the winter has been suggested to be associated with a higher burden of cardiovascular and pulmonary diseases among older patients during the winter months [18]. Furthermore, epidemiological data have demonstrated that peaks in seasonal influenza activity are associated with increases in AKI-related hospitalizations, suggesting that influenza epidemics may contribute to the seasonal variation in AKI incidence [37]. Consistent with this observation, population-based analyses have reported that influenza vaccination is associated with a reduced risk of AKI among older adults [38]. Similarly, during the COVID-19 pandemic, temporal analyses have shown that peaks in SARS-CoV-2 transmission were accompanied by increases in AKI incidence, suggesting that pandemic waves may influence the epidemiology of AKI [39]. AKI is a recognized complication of natural COVID-19 infection, particularly among hospitalized patients, and has been strongly associated with increased mortality [39,40]. Moreover, several observational studies have suggested that COVID-19 vaccination may be associated with a lower risk of AKI [41]. However, case reports have also described AKI following COVID-19 vaccination, and a recent study reported a higher incidence of AKI among individuals who received the vaccine [42]. The epidemiological pattern of AKI in tropical countries largely reflects infectious diseases as major underlying causes, such as acute gastroenteritis and malaria [43]. However, outside tropical and subtropical regions, evidence directly linking summer increases in AKI to specific acute illnesses, including infectious diseases, remains limited. Taken together, these findings underscore the complex and multifactorial relationship among infection burden, vaccination, and AKI risk.

In addition, seasonal changes in host-related factors of lifestyle and behavioral patterns—including dietary habits (e.g., fruit, vegetable), occupational heat exposure (e.g., outdoor labor), physical activity and sports participation, and seasonal variation in medication use—may also contribute to changes in renal hemodynamics and susceptibility to AKI. For example, studies of young workers engaged in physically demanding occupations such as sugarcane harvesting have reported episodes of acute kidney injury during the harvest season, which are thought to be related to heat stress, dehydration, and intense physical exertion [44,45]. Because seasonal variation in AKI is influenced by multiple factors, a single uniform preventive strategy may be difficult to implement. However, because outpatient AKI is often mild and potentially reversible, recognizing seasonal risk patterns may provide an opportunity for early intervention and prevention before progression to more severe kidney injury. From a preventive perspective, these findings underscore the importance of patient education in the outpatient setting, including enhanced seasonal monitoring of renal function, careful review of medication use, reinforcement of hydration counseling during periods of extreme temperatures, and appropriate vaccination prior to epidemic seasons. In particular, a summer-associated increase in outpatient AKI risk was observed among patients aged 65–74 years and those with CKD stages 2–3, suggesting that relatively active individuals with preserved or moderately impaired kidney function may be more vulnerable to seasonal stressors.

Several limitations of this study should be acknowledged. First, the incidence of outpatient AKI observed in this study was lower than that reported in previous studies of outpatient AKI. This is likely attributable to the exclusion of patients who were hospitalized, initiated dialysis, or experienced short-term AKI events, which may have led to underestimation of AKI episodes. Some acute and more severe episodes may have been missed, potentially influencing not only the overall magnitude but also the seasonal distribution and severity profile of outpatient AKI observed in this study. Second, baseline kidney function and AKI episodes were defined based on routinely collected laboratory data. Detailed clinical information—including symptoms, blood pressure, medication use and adherence, the exact timing of AKI onset, and acute intercurrent illnesses—as well as environmental and behavioral data, such as fluid intake, dietary patterns, individual-level exposure to ambient temperature, and behavioral adaptations (e.g., air conditioner use), were not available. These environmental and behavioral factors themselves are subject to seasonal variation and may have influenced the observed seasonal patterns of outpatient AKI. In addition, although comorbid conditions such as hypertension, diabetes mellitus, cardiovascular disease, and malignancy are major determinants of AKI risk, detailed information regarding these comorbidities was not fully captured in our dataset. Therefore, residual confounding related to these clinical factors cannot be excluded. Further prospective studies incorporating comprehensive clinical, comorbidity, and environmental data are required to more accurately characterize seasonal variations in outpatient acute kidney injury. Third, the study population was predominantly composed of nephrology outpatients with underlying CKD at a single institution, with more than 80% of the cohort having an eGFR < 60 mL/min/1.73 m^2^. In a population with a high prevalence of CKD, fluctuations in kidney function may reflect not only true acute kidney injury but also progression of the underlying chronic kidney disease or hemodynamically mediated reversible changes, such as transient reductions in blood pressure or dehydration associated with diuretic use. Therefore, the observed associations may, at least in part, be influenced by these factors, and the findings may not be generalizable to a broader general outpatient population. Fourth, the definition of outpatient AKI in this study differs from the KDIGO criteria in terms of the specified time window. Because we adopted a 14–90 days interval rather than the 48h to 7-day window defined by KDIGO, some events may represent subacute or chronic kidney function decline rather than strictly defined acute kidney injury. Because this time frame was arbitrarily determined, it may have introduced bias and resulted in potential misclassification.

## 5. Conclusions

This large observational study conducted in a nephrology outpatient setting demonstrated that the risk of outpatient AKI increases during both the summer and winter seasons among patients with CKD, with distinct seasonal patterns according to AKI severity. The seasonality of outpatient AKI may be associated with multiple contributing factors, including the seasonal distribution of underlying acute illnesses and temperature-related physiological changes affecting renal hemodynamics. Recognition of these stage- and season-specific patterns may help identify vulnerable populations and inform targeted preventive strategies for outpatient AKI across different seasons.

## Figures and Tables

**Table 1:** Baseline Characteristics of the Study Population. CKD: chronic kidney disease; eGFR: estimated glomerular filtration rate.

Total Number	*n* = 203,853	*n* (%)
Sex		
male	117,490	57.6%
female	86,363	42.3%
Age category (years)		
<65 years	94,056	46.1%
65–74 years	62,166	30.4%
≥75 years	47,631	23.3%
CKD stage		
Stage 2 (60 ≤ eGFR < 90)	76,215	17.7%
Stage 3 (30 ≤ eGFR < 60)	91,544	44.9%
Stage 4 or 5 (eGFR < 30)	36,094	37.3%

## Data Availability

The data presented in this study are available on request from the corresponding author.
